# Primary intracranial malignant melanoma in an adolescent girl with NRAS and TP53 mutations: case report and literature review

**DOI:** 10.3389/fonc.2024.1465676

**Published:** 2024-11-22

**Authors:** Xinyu Liu, Hailiang Shi, Xiaolong Wen, Kuo Zhang, Ge Feng, Jie Wei, Hebo Wang

**Affiliations:** ^1^ Graduate School of North China University of Science and Technology, Tangshan, Hebei, China; ^2^ Hebei General Hospital, Shijiazhuang, Hebei, China; ^3^ Graduate School of Hebei Medical University, Shijiazhuang, Hebei, China; ^4^ Hebei Provincial Key Laboratory of Cerebral Networks and Cognitive Disorders, Shijiazhuang, Hebei, China

**Keywords:** case report, primary malignant melanoma, pediatric, adolescent, seizures, nevus, NRAS

## Abstract

Primary intracranial malignant melanoma(PIMM) is often difficult to treat in patients without a history of skin melanoma or extensive melanin deposition. Due to the rarity of the disease, the current accepted treatment is surgical resection, but the prognosis is still poor. We report a case of PIMM in an adolescent girl with epilepsy as the only symptom and atypical imaging findings. PIMM was confirmed by further pathological and clinical examination. We summarize previous cases to discuss the clinical manifestations, imaging, pathological and genetic characteristics of the disease, aiming to improve the clinician’s understanding of the disease. This case underscores the PIMM as a differential diagnosis and prompt surgical treatment for adolescents with epileptic seizures accompanied by intracranial space-occupying lesions, even in the absence of extensive skin blackening.

## 1 Introduction

Primary intracranial malignant melanoma (PIMM) is one of the four primary melanocytic tumors identified in the 2016 WHO Classification of Central Nervous System Tumors (1). It is uncommon, accounting for approximately 1% of all melanoma cases and 0.07% of all brain tumors (2). Melanin-producing melanocytes in the human body are derivatives of ectodermal neural crest cells and can therefore be produced at sites of neural crest migration during embryonic development, such as the skin, heart valves, inner ear, and pia and uvea of the eye (3–5). Intracranial melanomas usually metastasize from cutaneous malignant melanomas or are associated with neurocutaneous melanomas (NCM). Owing to the rarity of the disease, there is no standardized treatment protocol, and complete surgical resection is the currently accepted treatment. Adult survival after surgery can reach 17 years, whereas pediatric patients have a poor prognosis, with a median survival of only 8 months (6, 7). Here, we report the case of an adolescent girl who presented with seizures that were pathologically and clinically diagnosed as PIMM with neuroblastoma RAS viral oncogene *(NRAS)* mutations.

## 2 Case descriptions

**Figure 1:**
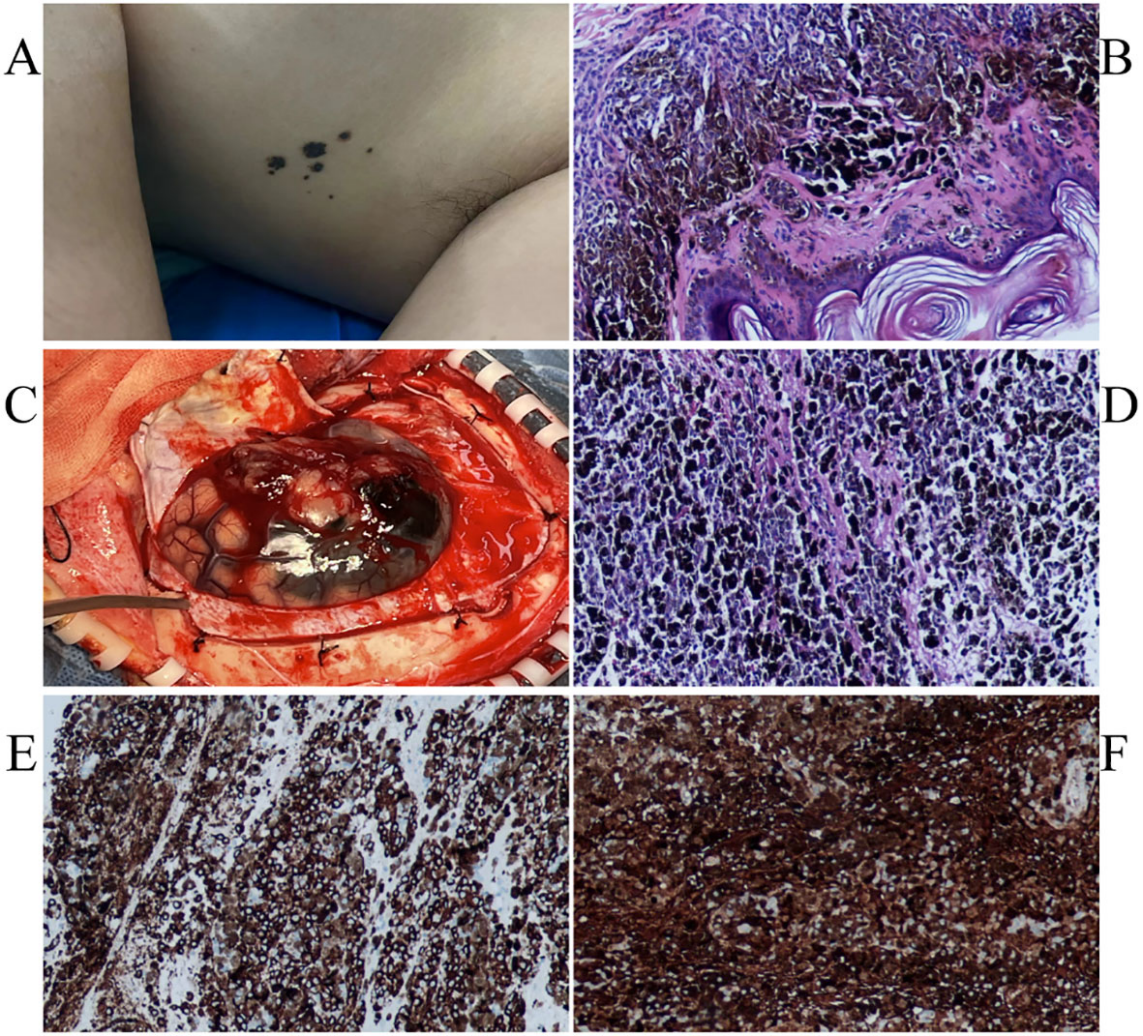
Several small speckled black pigmentations were seen in the left axilla **(A)**. The pathological findings of skin nevus suggested benign intradermal nevus **(B)**. Intraoperatively seen **(C)**.The immunohistochemical staining: HMB45 + **(D)**, S100 + **(E)**.The pathological findings of skin nevus suggested benign intradermal nevus **(F)**.

The patient was a Chinese girl, 17 years old, presenting mainly with loss of consciousness and tonic convulsions of the limbs, with no obvious abnormalities on neurological examination. Computed Tomography (CT) scan revealed an irregular and high-density mass in the right frontal region with clear borders and curved edges of approximately 45×26 mm (**Figure 1A**). Magnetic resonance imaging(MRI) showed low T1WI signal along with T2WI signal, and other signals in the right frontal area. A high signal on T1WI and low signal on T2WI were observed around the lesion. The maximum transverse section was approximately 25 × 30 mm, edema signal images could be seen around the lesion, scan enhancement was significantly uneven and enhanced, and the adjacent meninges could be enhanced. Perfusion in the lesion area was higher than that in the contralateral area; the Cr peak was lower, the Cho peak was higher, the NAA peak was not observed, and the Cho/Cr ratio was higher (**Figures 1B-F**).

**Figure 2:**
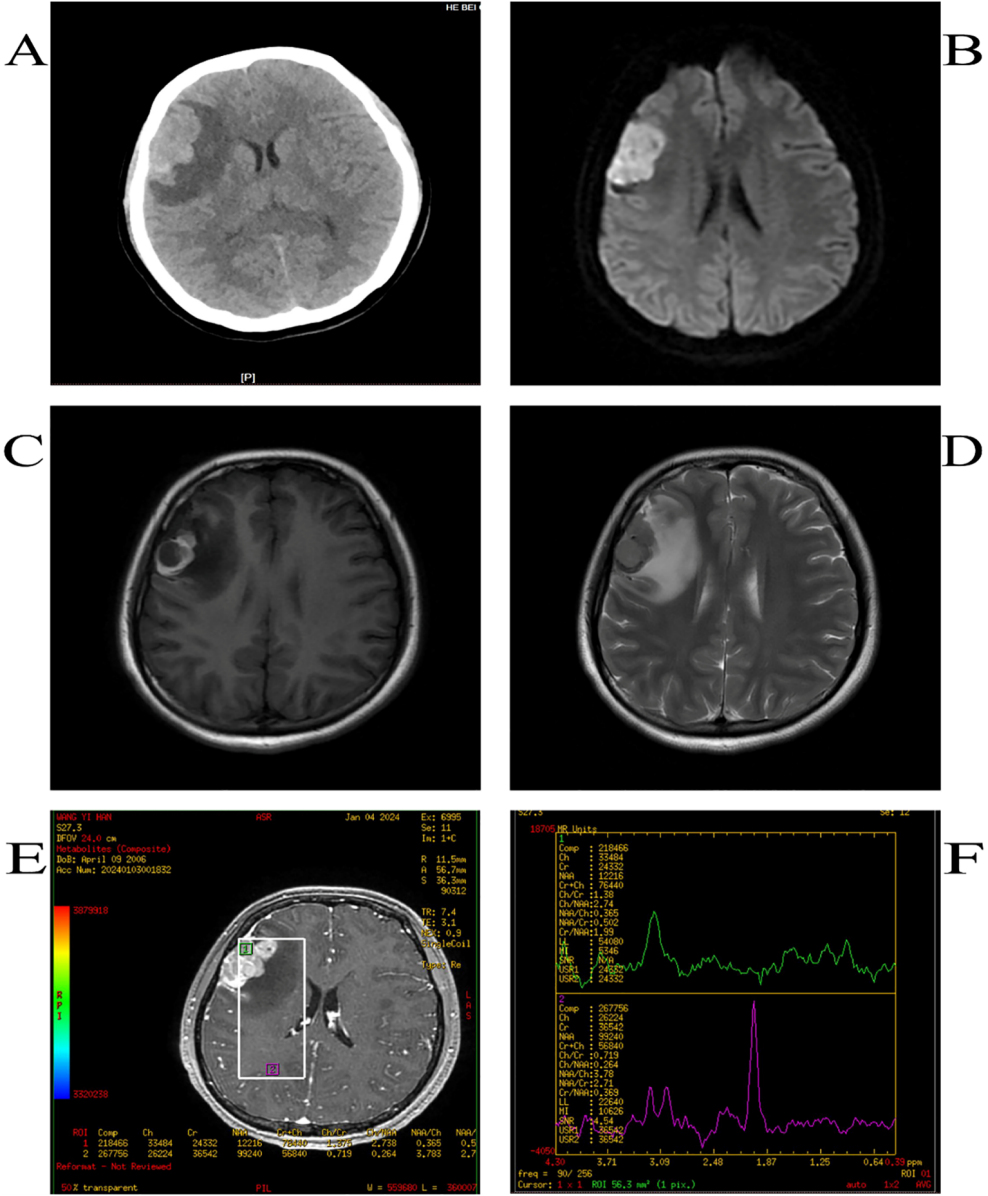
CT shows high-density opacities such as round-like images **(A)**,DWI high signal **(B)**,T1WI high-signal **(C)**, T2WI low-signal shadow **(D)**, the Cr peak was lower, the Cho peak was higher, the NAA peak was not shown, and the Cho/Cr was higher **(E, F)**.

General examination revealed a small amount of mottled black pigment in the left armpit, which her mother reported had been present at birth and gradually increased with age. The current diameter was 0.1-0.5 cm (**Figure 2A**). Subsequently, cutaneous nevus resection was performed under local anesthesia, and the pathological results indicated a benign intradermal nevus (**Figure 2B**). No abnormal melanin deposition was observed in other parts of skin, mucous membranes, or during eye examinations.

The patient underwent total craniotomy to remove the brain tumors. During surgery, the tumor was located above the lateral fissure, with some black and yellow-white tumors located in the subarachnoid space. There was no obvious dividing line between the tumor and brain tissue, and the tumor size was approximately 4×3×2 cm (**Figure 2C**).A common PIMM appears black to the naked eye. In this case, the tumor was partly black and partly yellowish-white, which is an unusual pattern of melanin distribution. This suggests that even if the tumor is not completely black, it is important to suspect the possibility of PIMM in time. When observed under the microscope, the tumor was composed of pleomorphic tumor cells arranged in a solid sheet, with obvious cell atypia, a large number of coarse brown and black pigment particles in the cytoplasm, a large nucleus, an obvious nucleolus, and mitotic images. Immunohistochemical staining revealed vimentin (+), S100 (+), HMB45 (+), Melan A (+), Ki-67 (approximately 70%+), and nestin (+) expression, consistent with malignant melanoma (**Figures 2D–F**).

**Figure 3:**
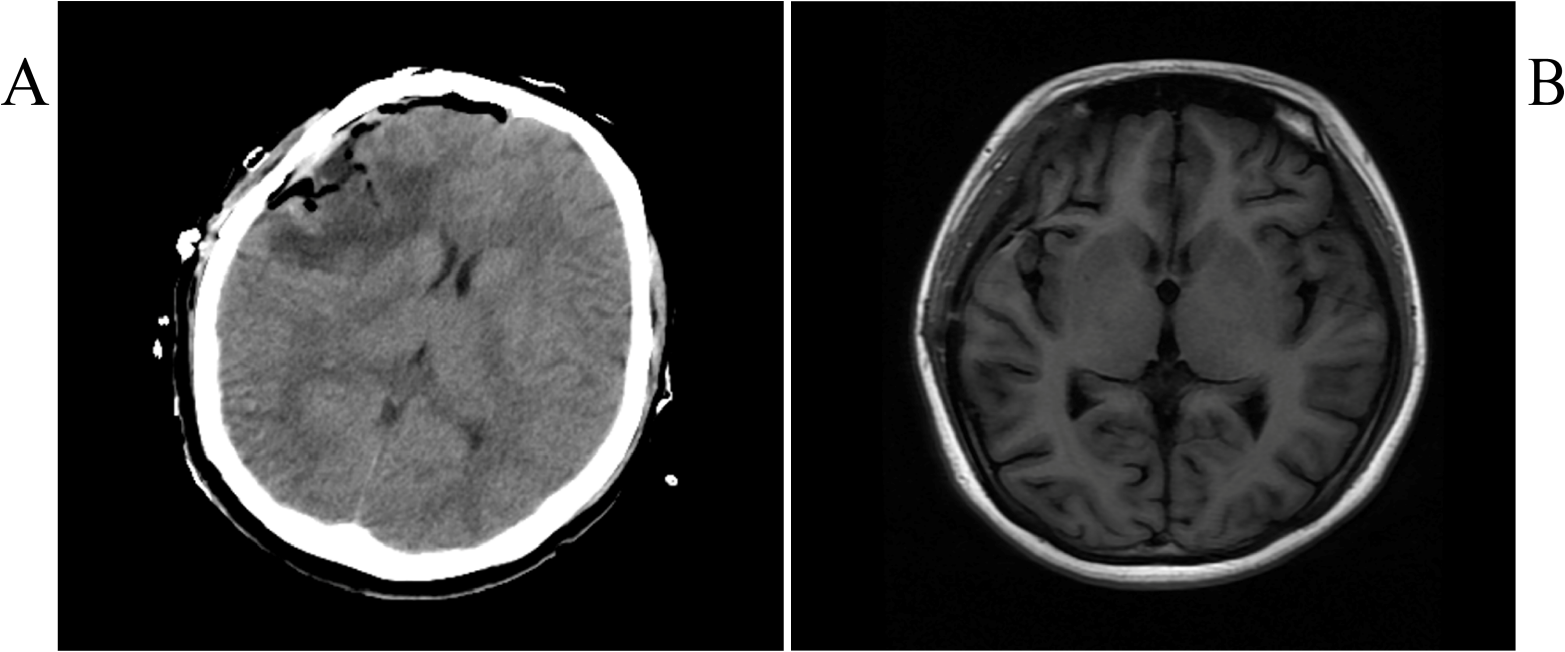
Postoperative CT **(A)** and MRI **(B)**.

Postoperative head CT and MRI showed complete resection (**Figures 3A, B**), along with lymph node examination, abdominal pelvic CT-enhanced scan, and whole-body bone imaging showed no significant abnormalities. Further examinations by dermatologists and ophthalmologists revealed no suspicious skin and uveal melanoma.

After surgery, with the consent of the patient’s family, the intracranial melanoma resected during surgery was subjected to genetic testing, and the results were positive for *NRAS c.181C>A,p.Gln61Lys*; positive for *TP53 c.743G>A,p.Arg248Gln*; and negative for *BRAF* mutation (entrusted to the Yixian Medical Laboratory), which has been reported to be pathogenic in the literature (6, 7).

The patient was discharged from the hospital on the 13th day after surgery with no discomfort. Afterwards, she was admitted to the oncology department and received radiotherapy (brainstem<3500cGy, optic nerve<2500cGy, lens<500cGy, pCTV prescription dose: DT42Gy/4.2 Gy/10f). NRAS-mutant PIMM has no effective targeted drug therapy. Toripalimab is a fully human monoclonal antibody against the PD-1 receptor. It can bind to PD-1 on the surface of T lymphocytes and block its binding to PD-1 ligands on tumor cells, so that the suppressed T cells can recover the recognition function of tumor cells and achieve antitumor effects. This has been approved for the systemic treatment of unresectable or metastatic melanoma. PIMM has a poor prognosis and is prone to metastasis and recurrence. To prevent the metastasis and recurrence of the tumor, improve the prognosis, and keep the drug affordable for the patient’s family, she was treated with Toripalimab. After 5 months of follow-up, no discomfort such as tetanic limb convulsions occurred.

## 3 Materials and methods

### 3.1 Search strategy

Two authors (XL and XW) independently conducted a literature search of PubMed, EMBASE, OVID, Web of Science, and CNKI databases from their inception to June 17, 2024, using the following keywords: children AND intracranial malignant melanoma.

### 3.2 Study selection and analysis

**Figure 4:**
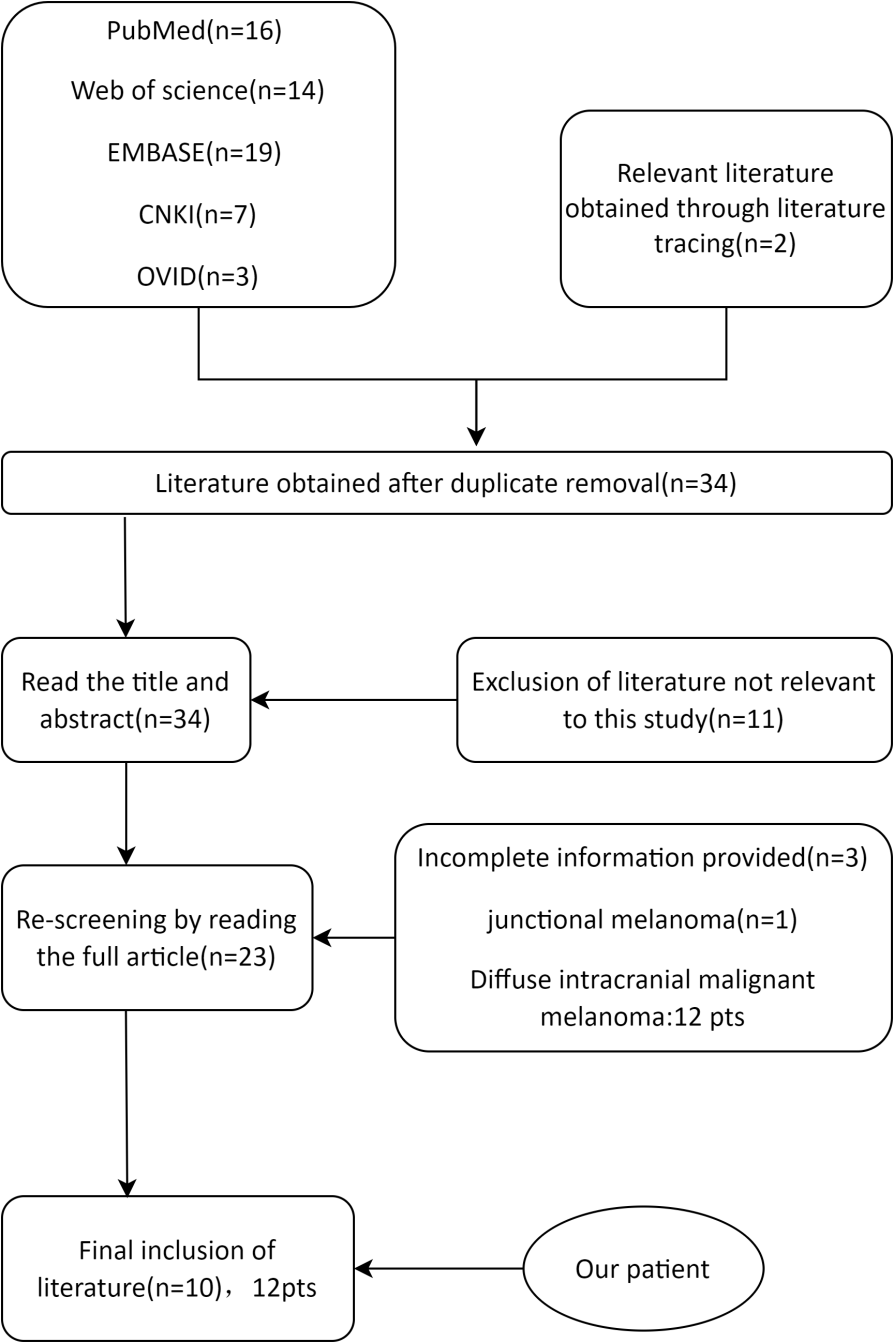
Screening process for past cases.

Patients younger than 18 years with a pathological diagnosis of PIMM were selected. This study focused on isolated PIMM, similar to the present case; therefore, patients with diffuse meningeal changes were excluded. After reading the titles, abstracts, and full texts of the publications, irrelevant literature and cases were excluded (**Figure 4**). All data were statistically analyzed using IBM SPSS Statistics 27.0. Measurements that conformed to normal distribution were expressed as mean ± standard deviation (x ± SD). Data that were not normally distributed were expressed as medians (25%, 75%). Count data are expressed as n (%).

## 4 Results

A total of 59 literatures were retrieved. After removing the duplicates, 42 articles were identified. After the initial screening and re-screening, 10 studies met the inclusion criteria (**Table 1**). The final study cohort consisted of 11 patients from 10 literatures (7–16). Demographic and clinical characteristics of the patients were analyzed. In recent years, the number of reported cases of PIMM in children has been on the rise, with a median age of onset of 14 ± 3.19 years for all patients and an age range of 7-17 years. Surprisingly, except for a 7-year-old girl reported in 1993 (8), all cases involved adolescents aged 10-17 years. 50.00% (6/12) of the patients in this study cohort were male, with a male to female sex ratio of 1:1. Of the 12 cases of primary intracranial malignant melanoma in children, headache occurred in 10 (83.33%), epilepsy in 5 (41.67%), neurological impairment in 4, supratentorial tumors in 10 (83.33%), and congenital melanocyte nevi (CMN) in 6 (50.00%). Only the case reported in 2012 had obvious pigmented skin nevi (9), and the remaining cases had small CMN (diameter of approximately 1 cm or less). The vast majority of cases present with headaches, a few have seizures, and epilepsy as the sole presenting symptom is particularly rare (an earlier report and our case). Although almost all cases are treated surgically, 75.00% of the patients die because of the rapid progression of the disease. We suspect that adolescents with intracranial space-occupying lesions, even if the CMN is small, should be aware of the possibility of PIMM. Since most melanomas are *BRAF* mutations, *NRAS* mutations are rare, and not all cases have been analyzed for genetic testing; the information available is limited. **Lim et al.** reported the case of a 15-year-old boy with PIMM and an *NRAS* mutation who died 8 months later due to tumor recurrence despite radiation therapy and immunotherapy (15). Mondal et al. reported a case of PIMM in an 11-year-old girl who underwent surgical removal and postoperative radiotherapy, with a postoperative survival of more than two years (10).

**Table 1:** Characteristics of intracranial malignant melanoma in children.

Num	Year	Age/Sex	Symptoms	Tumor location	CMN (yes/no)	exairesis	Treatment	Survival (month)
1	1993 (8)	7/F	headache	Right frontal lobe	no	yes	RT,CT	Died at 7month
2	2012 (9)	13/M	headache vomiting seizures	right frontal region	yes	yes	RT,CT	Died at 7month
3	2015 (10)	16/M	headache, convulsions, blurring of vision, and vertigo	left temporal	yes	yes	RT,CT	Died at 7month
4	2016 (11)	11/F	Headache, vomiting, ataxia, right temporal hemianopia	left occipital region	yes	yes	RT	Over 2 years
5	2019 (12)	16/M	headache, vomiting, Left limb weakness.	right parietal region	no	yes	RT	Died 9 months
6	2020 (13)	16/F	headache, dizziness, vomiting.	Left temporal	no	yes	RT,CT	Died at 2.5 months
7	2020 (13)	11/M	headache, vomiting.	Left temporal	yes	yes	RT	Died at 3months
8	2021 (14)	17/M	seizures	right-frontal-lobe	yes	yes	RT, nivolumab and ipilimumab	Died at 13 month
9	2022 (15)	12/F	headache vomiting	left skull base, right amygdala, multiple small tumors on the cerebral, brain stem and spine	no	yes	CT, nivolumab	Died at 4 month
10	2023 (16)	15/M	headache vomiting	right frontal lobe	no	yes	RT, Nivolumab and Ipilimumab	Died at 8 month
11	2024 (17)	17/F	Headache, vomiting,left-sided facial palsy.	left basal ganglia	no	yes	no	died
12	2024	17/F	seizures	left frontal lobe	yes	yes	RT	Over 6 month

F, female; M, male; RT, radiotherapy; CT, Chemotherapy.

## 5 Discussion

PIMM is uncommon and even rarer in children and adolescents, with an increasing incidence in the last 20 years. Clinically, these patients present with headaches, focal neurological deficits, seizures, mental state alterations, and intracranial hypertension (17). Of the 12 patients aged < 18 years, 11 were adolescents aged 10-18 years. The vast majority of patients have headaches, approximately half have seizures, and in this case, epilepsy was the only symptom in very few patients.

Intracranial melanomas usually appear atypically on CT scans, mostly as round, high-density shadows, and are easily confused with cerebral hemorrhages, cerebral hemorrhagic cavernous malformations, meningiomas, and gliomas (1, 18). Owing to the para-magnetism of melanin, it is specific to MRI and is closely related to melanin content, which leads to different MRI manifestations. According to Isiklar et al., intracranial melanoma may have the following imaging patterns: typical melanoma with >10% melanocytes (high T1WI and low T2WI); amelanotic melanoma (low-or isointensity T1WI and high-or isointensity T2WI), indeterminate melanoma (mixed or does not meet the criteria for the first two types), and hematoma (MRI features of a hematoma only) (19). The MRI findings in our case did not show typical melanoma features, and it was confirmed after surgery that this was a mixed melanoma (**Table 2**). The tumors were partly black and partly yellowish-white, and the distribution of melanin was not uniform. These unique magnetic resonance findings are related to the unique distribution of melanin in tumors. Conventional MRI alone is insufficient to fully understand intracranial space-occupying lesions. Advanced MR techniques, including Diffusion-Weighted Imaging (DWI), Magnetic Resonance Spectroscopy (MRS), and MR perfusion (MRP) use functional, metabolic, hemodynamic, and cellular methods that combine sequences to distinguish between benign and malignant lesions (20). Malignancies typically present with diffusion-limited intensity, hyperintense DWI, and low ADC intensity, with MRP showing increased perfusion and lower NAA/Cho, NAA/Cr, lactate, and lipid peaks on MRS (20). Combined with the above imaging features, our patient was likely diagnosed with an intracranial malignancy before surgery. Often, it is difficult to make a definitive diagnosis based on imaging alone; the final diagnosis is based on pathological findings.

**Table 2:** Typical and atypical imaging features of PIMM.

MRI sequence	typical characteristic(>10% melanocytes)	amelanotic melanoma	our case
T1	high	low or isointensity	low in the middle and high around
T2	low	high or isointensity	isointensity

PIMM is rarely reported, and in most cases, melanoma involvement of the central nervous system is indicative of metastasis. As there are no significant differences in clinical presentation, imaging, and pathology between primary and metastatic intracranial melanomas, diagnosis is mainly based on the presence of extracranial melanomas (2). Typically, patients without extracranial primary lesions are diagnosed with primary intracranial melanoma. Patients with primary central nervous system melanoma have better clinical outcomes than patients with metastatic disease because of the possibility of long-term tumor control (21). Once ventricular metastases occur, the prognosis worsens, with a median survival of a few months, which is associated with difficulties in treatment approaches (17).

Similar to intracranial melanoma, simple cerebral hemorrhage, meningioma, and glioma can all lead to headache, increased intracranial pressure, and focal nerve function deficit. It can also appear as circular and other high-density shadows on CT, thus making challenging the differential diagnosis (22–24). Cerebral hemorrhage usually starts quickly, with headache, vomiting, and hemiplegia as the initial symptoms. Most patients have a history of hypertension and intracranial aneurysm and have different MRI signal characteristics according to different bleeding periods. The most common symptom of glioma is epilepsy. The stimulation of the cerebral cortex by the tumor causes abnormal discharges in the cortex, which can trigger epilepsy. Meanwhile, symptoms such as headache and vomiting indicating increased intracranial pressure may also occur. The tumors are mostly located in the white matter area or beneath the cortex. MRI shows a low T1 signal and a high T2 signal. High-grade tumors are often accompanied by cystic changes, necrosis, and obvious edema, with obvious uneven enhancement and a tendency to recur. Currently, surgery is the preferred treatment option. In cases of meningioma, CT shows equal density, with MRI showing equal T1 and T2 signals which is relatively uniform. Enhanced scan demonstrates significantly uniform enhancement, with a visible “meningeal tail sign”. Usually if the tumor is small (< 2 cm) and asymptomatic, it can be observed with follow-up visits. However, if progressive enlargement or clinical symptoms arise, surgery is required. [^68^Ga]Ga-DOTA-SSTR PET Radiotracers helps in the diagnosis and treatment of meningiomas (25).

Intracranial melanoma in children is often accompanied by neurocutaneous melanosis, with large (> 20 cm) or multiple (≥3) melanocyte moles at birth and benign or malignant melanocyte proliferation in the central nervous system (26). Ultraviolet exposure is an important risk factor for the development of nevi and cutaneous melanomas. In contrast, CMN, moles that form in the maternal womb and appear at birth, typically contain *NRAS* mutations and lack *BRAF* mutations, which typically occur in moles that form after birth (27). *NRAS* mutations have previously been reported in primary intracranial melanomas in children, but are rare in adults (15). Interestingly, Malin reported two cases of PIMM in children, both with oncogenic mutations of *NRAS* in the tumor; skin dysplasia manifested as congenital skin nevus without a skin tumor, and melanocytes in the pial meninges progressed to aggressive PIMM (28). Kinsler et al. found that different CMNS from patients with multiple CMN contained the same *NRAS* mutation, which was also present in the neuropathy of these patients. This suggests that multiple CMN and neuromelanism are caused by *NRAS* mutations, which probably emerged from the developing neural crest or neuroectoderm in the patient’s embryo (29). This finding is similar to that of our case. All the above studies suggest that central nervous system melanocytes are more susceptible to malignant proliferation by oncogenic *NRAS* than cutaneous melanocytes, but the reason remains unclear. This is similar to our case because CMN gene testing was not performed, but it is certain that the intracranial malignant melanoma in our case was caused by *NRAS* mutations. Combined with previous studies, it cannot be ruled out that the CMN and intracranial tumor tissues in our case were derived from nerve spines during embryonic development and had the same *NRAS* mutation, thus adding some information to the current limited experience.

## 6 Conclusion

In conclusion, it is almost impossible to diagnose PIMM based on clinical manifestations and imaging studies alone; the diagnosis of the disease is currently dependent on pathology. However, the present clinical experience can help us narrow this down, and our case adds some information to the limited clinical experience. When treating children with epilepsy as the main symptom accompanied by intracranial space occupying lesions, the possibility of intracranial melanoma should be considered in time for the presence of congenital melanocytic nevus, regardless of the size of the nevus. Early diagnosis and timely surgical intervention are necessary to treat this deadly disease.

## Data Availability

The data presented in the study are deposited in the Biological Project Library repository, accession number PRJNA789862.
